# Short-time thermal inactivation of surrogates of the public transport microbiome with a low-cost thermoresistometer

**DOI:** 10.1038/s41598-026-35087-3

**Published:** 2026-01-09

**Authors:** Henrik Grübbel, Yen Ly-Sauerbrey, Franca Arndt, Bruno Pavletić, Stefan Leuko, Frank Rinderknecht

**Affiliations:** 1 German Aerospace Center (DLR), Institute of Vehicle Concepts, Stuttgart, Germany; 2 German Aerospace Center (DLR), Institute of Aerospace Medicine, Cologne, Germany

**Keywords:** Thermal inactivation, Microorganims, Microbiome, Thermoresistometer, Short-time, Public transport, Diseases, Microbiology

## Abstract

In this study, the thermal inactivation of the bacterial genera *Staphylococcus*, *Enterococcus* and *Burkholderia*, which can be found in public transportation environments, as well as the bacteriophage MS2 as a surrogate for potential viral pathogens are investigated. To quantify the thermal inactivation characteristic, an automated and inexpensive thermoresistometer is constructed and set up, which enables the microorganisms to be exposed to short-term thermal shocks. The time dependent temperature curves were measured to account for heat-up and cooling times. Afterwards, the microorganisms were exposed to temperatures in the range of $50\,^{\circ }\hbox {C}$ to $85\,^{\circ }\hbox {C}$ for durations of 2 s up to 10 s and the thermal inactivation of the respective microorganisms was measured by counting colony forming units (CFU) and plague forming units (PFU). The data was visualized and fitted to an analytical thermodynamic model based on a first-order reaction and the Arrhenius equation to predict thermal inactivation times. This study reports the first measured thermal inactivation values for *E. viikkiensis* and *B. lata*, which have not been studied before. The results for MS2 and *S. capitis* show significantly shorter inactivation times than previous experiments. After exposure to $85\,^{\circ }\hbox {C}$ for 2 s there was no measurable survival of all tested microorganisms. The semi-automated test setup used allows for consistent measurements and can be adapted by other research groups.

## Introduction

Human passengers are the primary source of airborne and surface-bound microorganisms in public transport environments, which are introduced through skin contact, respiratory droplets or aerosols^1^. In addition to known pathogens such as SARS-CoV-2, several studies have characterized the microbiomes of indoor air and surfaces in transport systems, frequently identifying genera such as *Staphylococcus*, *Enterococcus*, *Sphingomonas*, and *Streptococcus*^2–4^. Of particular concern are the so-called ESKAPE pathogens: *Enterococcus faecium*, *Staphylococcus aureus*, *Klebsiella pneumoniae*, *Acinetobacter baumannii*, *Pseudomonas aeruginosa* and *Enterobacter* species. These bacteria are known for their ability to develop multidrug resistances and are primarily associated with hospital settings, but have also been found in public transport environments, likely due to frequent use by healthcare workers and patients^5–7^. To mitigate aerosol-based transmission in public transport, several countermeasures have been proposed, such as the use of high-efficiency particulate air (HEPA) filters^8^, increasing the fresh air supply^9^, or requiring passengers to wear masks^10^. An alternative approach is the thermal inactivation of potentially infectious aerosols. Heat can damage cell membranes, denature enzymes or degrade DNA/RNA, leading to microbial inactivation. This principle is already widely applied in food safety (e.g. pasteurization) and in sterilization processes in laboratories and medical facilities. While thermal inactivation is not yet established for aerosol disinfection, it has been proposed by various authors^11–13^. One of the main challenges of thermal inactivation is its high energy demand. However, in winter conditions, when cabin heating is already required, the existing thermal energy could be harnessed for microbial inactivation without significantly increasing energy consumption. Despite their small interior volumes, battery-electric public transport vehicles require high heating capacities, leading to significant energy consumption for climate control and reduced driving range in winter^14^. Compared to buildings, this makes thermal inactivation particularly promising for public transport environments.

To implement thermal inactivation effectively, a comprehensive understanding of the temperature-dependent inactivation kinetics of the target microorganisms is required. Microbial inactivation typically follows first-order reaction kinetics^15^, which can be described by:

1 $$\begin{aligned} \ln \left( \frac{C_t}{C_0}\right) = -kt \end{aligned}$$

where $C_t$ is the microbial concentration at time *t*, $C_0$ is the initial concentration, and *k* is the reaction rate constant. While the natural logarithm (ln) is used here, the term “log-linear model” in microbiology often refers to the same first-order behavior expressed with base-10 logarithms. The two forms differ only by a constant factor ($\ln 10$). This model has been widely applied to describe the thermal inactivation behavior of both viruses^16^ and bacteria^17^. First-order reaction kinetics are commonly employed to model thermal inactivation, but they may fail to capture complex patterns such as shoulder and tailing effects or variability within microbial populations^18^. The temperature dependency of *k* can be modeled using the Arrhenius equation^16^. Here, *A* is the frequency factor, $E_a$ the activation energy, *R* the universal gas constant, and *T* the absolute temperature:

2 $$\begin{aligned} k = A \exp \left( -\frac{E_a}{RT}\right) \end{aligned}$$

Accurate modeling of microbial inactivation requires experimental data regarding the thermal resistance of microorganisms commonly encountered in public transport environments. While previous studies have applied such models to experimental data, for example, on SARS-CoV-2^19^, data remain scarce for most microorganisms, particularly regarding short-term exposure to thermal shocks. For disinfection of vehicle cabin air, as well as for other applications such as fluid treatment, only short exposure times are available for microbial inactivation due to spatial and thermal constraints. A system capable of applying rapid, near-isothermal thermal shocks is required to assess microbial inactivation at discrete temperatures while avoiding bias from slow temperature transitions. Previous studies have employed various methods to investigate short-duration thermal inactivation. These include manually immersing glass capillaries into hot water baths^20^, using computer-controlled thermoresistometers with steam heating^21^, or mixing microbial solutions with pre-heated liquids and sampling at intervals^22,23^. Another approach involves flowing microbial suspensions through heated and cooled capillaries to simulate controlled thermal shocks^24^. Manual immersion is the simplest method for testing microbial resistance to thermal shock and has the advantage of requiring only relatively small quantities of microorganisms. However, its reproducibility is limited, particularly for short exposure times. In this study, we present a semi-automated thermoresistometer that retains the simplicity of manual immersion while significantly improving repeatability. This enables the systematic investigation of short-term thermal inactivation. Sealed glass capillaries filled with microbial suspensions are automatically immersed into a heated water bath and subsequently cooled by forced convection using ambient air. The proposed thermoresistometer can be easily constructed and operated by other research groups. Microbial reduction is quantified via dilution series and the plate count method. The organisms used in this study–*Enterococcus viikkiensis* DSM 24043$^T$, *Staphylococcus capitis* DSM 111179, *Burkholderia lata* DSM 23089$^T$, and the *Escherichia* bacteriophage MS2 DSM 13767–are all classified as Biosafety Level 1 (BSL-1) according to German regulations, allowing experiments to be conducted in a BSL-1 laboratory.

The experimental setup of the thermoresistometer is not directly applicable for use in public transportation environments but it provides a crucial data baseline of the thermal inactivation kinetics of relevant microorganisms, addressing current gaps in literature. Additional studies are needed to discuss the potential integration in public transport vehicles and to compare its benefits and limitations to currently used technologies like HEPA-filters. The findings are not limited to applications in public transportation and can be extended to estimate thermal inactivation in a variety of systems, including water and wastewater treatment processes and thermal disinfection strategies used in medical and healthcare environments.

To the best of our knowledge, this study is the first to examine the short-term thermal inactivation of *B. lata*, *E. vikkiensis*, and *S. capitis*. It also introduces a low-cost, semi-automated thermoresistometer designed to improve experimental accuracy compared to manual immersion approaches.

## Methods

### Microorganisms and growth media

As previously discussed, the following microorganisms in Table 1 were used in the experiment. The microorganisms were obtained from the Leibniz Institute DSMZ German Collection of Microorganisms and Cell Cultures (Braunschweig, Germany). The strain *Enterococcus viikkiensis* DSM 24043$^T$ was isolated from the air of a broiler processing facility in Finland. The strain *Staphylococcus capitis* DSM 111179 was isolated from surfaces on the International Space Station. They serve as surrogates for more pathogenic *Enterococci*, such as *Enterococcus faecium*, and *Staphylococci*, such as *Staphylococcus aureus*, both of which are members of the ESKAPE pathogens. The strain *Burkholderia lata* DSM 23089$^{T}$ was isolated from forest soil and represents the genus *Burkholderia*, which has also been detected in public transport environments^1,25^. The isolation place of the bacteriophage MS2 remains unknown. However, MS2 is a commonly used surrogate for more dangerous viruses such as SARS-CoV-2^26^ or the Norovirus^27^. The bacterial species (*E. viikkiensis, S. capitis, B. lata*) were grown for 18 h under constant shaking at 200 revolutions per minute (rpm) at $37 \,^{\circ }\hbox {C}$ on 2x Reasoner’s 2A (TEKnova) and Brain Heart Infusion medium (Sigma-Aldrich) as shown in Table 1. The MS2 phages were grown in the presence of host *Escherichia coli* DSM 5695 in NZCYM medium (Sigma-Aldrich). The host was allowed to grow until the exponential phase for two hours, when the infection started. After 18 hours of growth in liquid culture shaking at 125 rpm, the phages were precipitated with polyethylene glycol (PEG) and diluted in phosphate buffered saline (PBS) buffer (PBS: Na$_{2}$HPO$_{4}$ 7.0 g, KH$_{2}$PO$_{4}$ 3.0 g, NaCl 4.0 g, per Liter, pH 7.5). To determine the phage titer, a double-layered agar method with NZCYM was utilized as described by Kropinski et. al. 2009^28^. The cell number was adjusted to $10^5 - 10^6$ colony forming units (CFU)/plaque forming units (PFU) in phosphate buffered saline.

**Table 1:** Microorganisms used in the experiment.

Strain	Growth	Reference
*Burkholderia lata* DSM 23089$^T$	R2A $37 \,^{\circ }\hbox {C}$	Vanlaere et al., 2009^29^
*Enterococcus viikkiensis* DSM 24043$^T$	BHI $37 \,^{\circ }\hbox {C}$	Rahkila et al., 2011^30^
*Staphylococcus capitis* DSM 111179	R2A $37 \,^{\circ }\hbox {C}$	Sobisch et al., 2019^31^
*Escherichia* Phage MS2 DSM 13767	NZCYM $37 \,^{\circ }\hbox {C}$	Emslander et al., 2022^32^

### Thermoresistometer and determination of thermal resistance

**Fig. 1:**
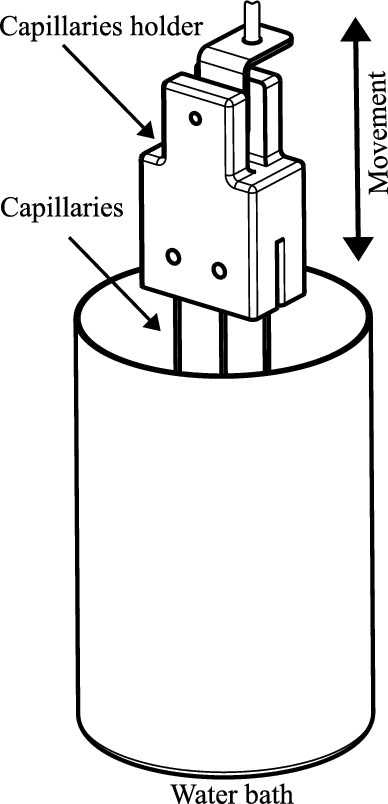
Functional principle of the thermoresistometer.

To expose the microorganisms to thermal shocks, glass capillaries were immersed in a temperature-controlled heating bath. Compared to conventional plastic test tubes, glass capillaries offer the advantages of lower wall thickness and reduced thermal mass, enabling faster and more uniform heat transfer. The capillaries were secured within a clamping block, which could be precisely lowered into and withdrawn from the heating bath via a linear guide mechanism. A schematic representation of the mode of operation is shown in Fig. 1.

**Fig. 2:**
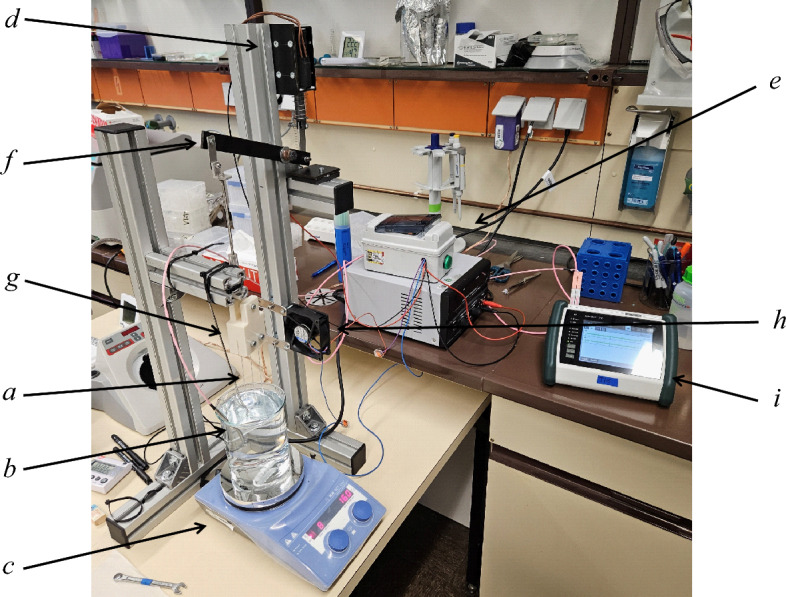
Experimental setup of the thermoresistometer for controlled thermal exposure; *a* 50 $\mu$L capillary; *b* water bath; *c* hotplate stirrer; *d* solenoid; *e* timing relays; *f* capillary holder; *g* rocker arm; *h* cooling fan; *i* datalogger.

To determine the thermal resistance in the experiment, the glass capillaries (*a*; HIRSCHMANN ringcaps REF 9600150, Germany) were filled with 50 $\mu$L of microbial solution with an initial CFU/PFU of $10^5 - 10^6$ using a pipette controller for capillaries (BRAND pipette controller micro, Germany). The glass capillaries were sealed using a flame and it was verified that the sealing had no significant influence on the viable count of the microorganisms. As a control, two samples of each microorganism were sealed in a glass capillary, stored in it during the experiments and then analysed together with the thermally treated samples via plate count. Heat treatment was done by submerging the class capillaries in a temperature controlled water bath (*b*) using a magnet hotplate stirrer (*c*; IKA rct basic, Germany) with a PT100 probe. The magnetic stirrer was set to 160 rpm to improve heat transfer and prevent thermal stratification of the water. By using a solenoid (*d*; Intertec ITS-LS-4035, Germany) and a digital timing relays (*e*; Crouzet DZ1R, France) operated with an 24 V laboratory power supply the submerging was automated for repeatability. The solenoid is connected to a rocker arm with a linear guide (*f*), which in turn is linked to a 3D-printed holder (*g*) that clamps the capillaries at their upper end. After the programmed exposure time, the glass capillaries are retracted to their original position by spring force and removed from the water bath. The full submerging of the glass capillaries takes approximately 0.23 s and the removal takes approximately 0.16 s, both measured by frame by frame analysis of a 30 fps video. When not submerged, the glass capillaries are constantly cooled with ambient air by a 24 V fan (*h*; ebmpapst 8414 NH, Germany). The temperature of the water bath is constantly monitored during the experiments by a datalogger (*i*; Ahlborn Almemo 710, Germany) and a type N thermocouple (*j*; Electronic Sensor, Germany). Compared to manual immersion of the glass capillaries, this experimental setup allows greater repeatability. Due to the standard components used, the setup is simple and inexpensive. The actual setup used in the experiments is shown in Fig. 2.

**Fig. 3:**
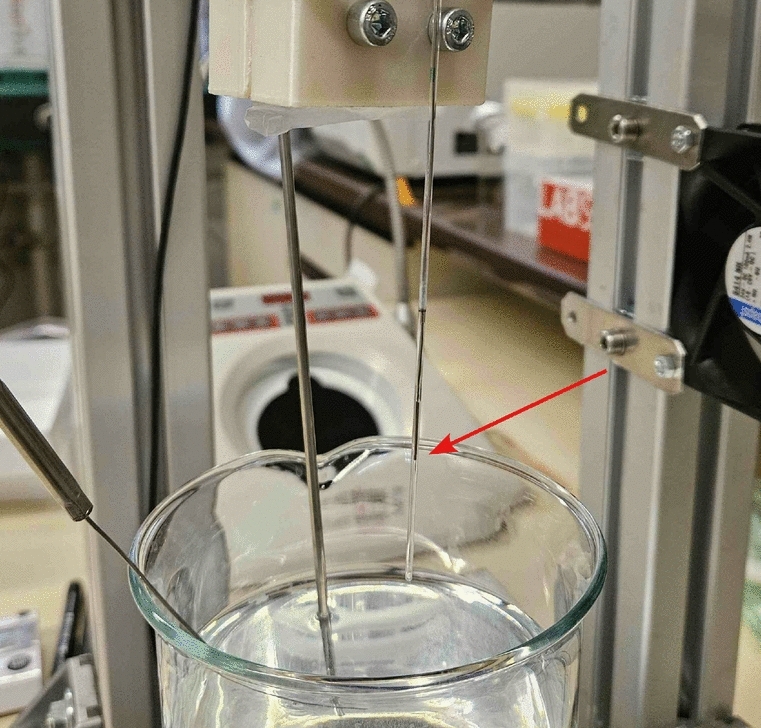
Type N thermocouple in glass capillary for temperature curve measurement.

In order to quantify and verify the thermal stress to which the microorganisms were exposed, the temperature curve was measured using a 0.5 mm thermocouple type N, which was inserted into a glass capillary filled with water. The temporal resolution of the measurement was 0.1 s. The thermocouple inside the glass capillary is shown in Fig. 3.

After the heat exposure, the glass capillaries were opened with a diamond needle file and the samples were retrieved. To determine the survival, a 10-fold dilution series from $10^{-1}$ to $10^{-8}$ was prepared and plated on respective media plates. After incubation at $37\,^{\circ }\hbox {C}$ for 48 h, colonies were counted and CFU/ml or PFU/ml were calculated. To determine the phage titer, a double-layered agar method with NZCYM was utilized as described before.

All experiments were done in technical triplicates. The decimal reduction times (D-value) were calculated according to the following equation:

3 $$\begin{aligned} D=\frac{t}{log_{10}N_0 - log_{10}N} \end{aligned}$$

Where *t* is the exposure time, $logN_0$ the logarithmic initial cell concentration and *logN* the logarithmic final population. The D-value represents the time required to achieve a one-log (10-fold) reduction in the number of viable microorganisms under specified conditions.The results were analyzed using Microsoft Excel 2019 for general data handling and R 4.5.1 for statistical evaluation. To assess the significance of the findings, an analysis of variance (ANOVA) was conducted, followed by a Tukey HSD post hoc test, using a significance level of 5 %.

### Experimental procedure

First, the thermal load on the samples was assessed by measuring the temperature inside the capillary, as previously described. Three target temperatures $60\,^{\circ }\hbox {C}$, $70\,^{\circ }\hbox {C}$, and $85\,^{\circ }\hbox {C}$ were selected for analysis. Subsequently, the thermal shock resistance of the microorganisms *E. viikkiensis*, *B. lata*, *S. capitis*, and MS2 was evaluated at varying exposure times and temperatures, as outlined in Table 2. Additionally, the temperature of $50\,^{\circ }\hbox {C}$ was tested for MS2.

**Table 2:** Temperatures and time intervals investigated in the experiments.

Microorganism	Timepoints			
	$50\,^{\circ }\hbox {C}$	$60\,^{\circ }\hbox {C}$	$70\,^{\circ }\hbox {C}$	$85\,^{\circ }\hbox {C}$
*Enterococcus viikkiensis*	-	4 s, 6 s, 8 s	2 s, 3 s, 4 s	2 s, 3 s, 4 s
*Staphylococcus capitis K1*	-	6 s, 8 s, 10 s	2 s, 3 s, 4 s, 6s	2 s, 3 s, 4 s
*Burkholderia lata*	-	2 s, 4 s, 6 s	2 s, 3 s, 4 s	2 s, 3 s, 4 s
MS2	3 s, 5 s, 7 s	3 s, 4 s	2 s, 3 s, 4 s	2 s, 3 s, 4 s

## Results

### Temperature measurements

**Fig. 4:**
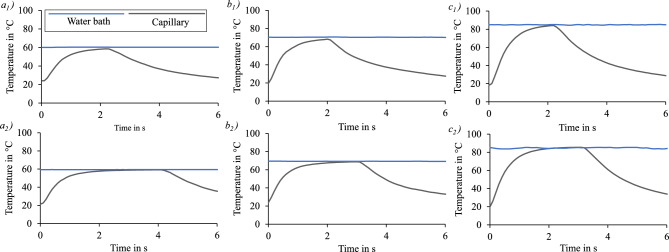
Temperature of the water bath (blue) and the buffer medium in the glass capillary (grey) when submerged in water bath with the set temperature of $60\,^{\circ }\hbox {C}$ for 2 s $(a_1)$ and 4 s $(a_2)$, $70\,^{\circ }\hbox {C}$ for 2 s $(b_1)$ and 3 s $(b_2)$ and $85\,^{\circ }\hbox {C}$ for 2 s $(c_1)$ and 3 s $(c_2)$.

Figure 4 presents the results of the temperature measurements. The blue curve shows the measured temperature of the water bath and the grey curve the measured temperature of the buffer medium in the capillary submerged and extracted from the water bath. The graph starts at $t=0\,s$ with the submersion of the capillary. The data obtained at $60\, ^{\circ }\hbox {C}$ (2 s, 4 s), $70\,^{\circ }\hbox {C}$ (2 s, 3 s), and $85\,^{\circ }\hbox {C}$ (2 s, 3 s) demonstrate that the heating and cooling of the glass capillary are not instant due to the thermal mass. As expected, this prevents the achievement of an ideal thermal shock. The temperature curves exhibit a rapid initial increase, which gradually levels off before reaching the target temperature. This behavior is attributed to the decreasing temperature gradient between the heating medium and the sample. The target temperature is typically reached after approximately 2 s. Cooling occurs rapidly through forced convection. For example, following 1 s of cooling at a bath temperature of 60 °C, the sample temperature drops to approximately 40 °C. At a bath temperature of 70 °C, this takes about 1.5 s, while at 85 °C, around 2 s are required.

A thermal tolerance was defined to take into account the heating and cooling time. The tolerance limit was defined as 5 % of the target temperature in $^{\circ }\hbox {C}$, so that only the time during which the sample reached at least 95 % of the target temperature in $^{\circ }\hbox {C}$ is counted as exposure time. Therefore the exposure time is calculated as follows:

4 $$\begin{aligned} t_{exp}=t_{set}+\Delta t \end{aligned}$$

Where $t_{exp}$ is the exposure time of the microorganism after the correction, $t_{set}$ is the set time of the submersion in the water bath and $\Delta t$ the time correction, which is calulated as follows:

5 $$\begin{aligned} \Delta t = t_{1}\left( \vartheta >0.95\vartheta _{set} \right) -t_{2}\left( \vartheta <0.95\vartheta _{set}\right) \end{aligned}$$

Where $t_{1}$ is the time when the probe reached 95 % and $t_{2}$ the time when the temperature $\vartheta$ falls below 95 % of the set exposure temperature $\vartheta _{set}$ in $^{\circ }\hbox {C}$. This calculation was carried out for the considered times and temperatures respectively. The mean value of the time deviation is $\Delta t=-1.3\,s$. As this value was derived directly from the measured sample temperature, the duration of immersion and removal of the sample are already included.

### Thermal inactivation of microorganisms

#### *Enterococcus viikkiensis*

**Fig. 5:**
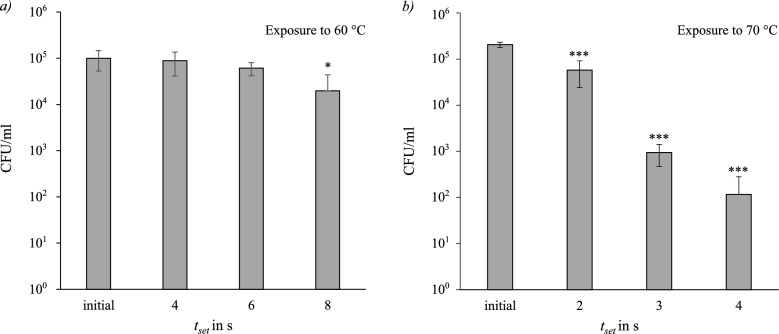
Survival of *Enterococcus viikkiensis*; *a)* after exposure to $60\,^{\circ }\hbox {C}$ for a set exposure time of (4 s, 6 s, 8 s); *b)* after exposure to $70\,^{\circ }\hbox {C}$ for (2 s, 3 s, 4 s). Significance level compared to initial (ANOVA): *$p\le 0.05$; ***$p \le 0.001$.

*Enterococcus viikkiensis* was tested at temperatures and set exposure times of $60\,^{\circ }\hbox {C}$ (4 s, 6 s, 8 s), $70\,^{\circ }\hbox {C}$ (2 s, 3 s, 4 s) and $85\,^{\circ }\hbox {C}$ (2 s, 3 s, 4 s). At $85\,^{\circ }\hbox {C}$ there was no measurable survival detected. The resulting concentrations in CFU/ml are displayed as bars in Fig. 5 in dependence of the exposure time and temperature. The measurements were performed in triplicates and the error bars show the standard deviation of each sample (n=3). The initial CFUs differ as the experiments were conducted on different days. At $60\,^{\circ }\hbox {C}$ the rate of inactivation increases with extended exposure time. The calculated D-value at $t_{set}=4\,s$ is $D_{4s,60 ^\circ C}=51.68\,\textrm{s}$ and decreased to $D_{8\,s,60\,^\circ C}=9.49\,\textrm{s}$ with $t_{set}=8\,\textrm{s}$. The D-values were calculated with the mean of the 3 samples and the corrected actual exposure time $t_{exp}$. After 8 s of exposure, thermal inactivation reaches approximately 80 %. According to the ANOVA analysis, this reduction is statistically significant $(p = 0.045)$. At $70\,^{\circ }\hbox {C}$ the rate of inactivation increases between $t_{set}=2\,\textrm{s}$ and $t_{set}=3\,\textrm{s}$ with with D-Values of $D_{2\,\textrm{s},70\,^\circ C}=1.28$ and $D_{3\,\textrm{s},70\,^\circ C}=0.73\,\textrm{s}$. After 4 s of set exposure time the inactivation is estimated at 99.9 %. At $70\,^{\circ }\hbox {C}$ the thermal inactivation was significant after all durations $(p_{2\,\textrm{s}}=0.00022, p_{3\,\textrm{s}}=0.00002, p_{4\,\textrm{s}}=0.00002)$. However, the differences between the thermally treated microorganisms at $70\,^{\circ }\hbox {C}$ were not significant.

#### *Staphylococcus capitis*

**Fig. 6:**
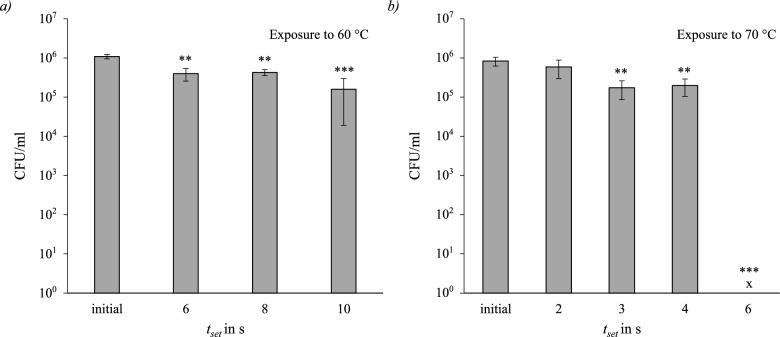
Survival of *Staphylococcus capitis*; *a* after exposure to $60\,^{\circ }\hbox {C}$ for a set exposure time of (6 s, 8 s, 10 s); *b* after exposure to $70\,^{\circ }\hbox {C}$ for (2 s, 3 s, 4 s, 6 s). Significance level compared to initial (ANOVA): **$p\le 0.01$; ***$p \le 0.001$.

*Staphylococcus capitis* was tested at temperatures and set exposure times of $60\,^{\circ }\hbox {C}$ (6 s, 8 s, 10 s), $70\,^{\circ }\hbox {C}$ (2 s, 3 s, 4 s, 6 s) and $85\,^{\circ }\hbox {C}$ (2 s, 3 s, 4 s). Compared to the previous tests, the exposure times were adjusted due to the higher thermal resistance. The concentration results are shown in Fig. 6, using the same format as previously. Again as with *E. viikkiensis* at $85\,^{\circ }\hbox {C}$, there was no measurable survival detected. The data show some inconsistencies, with a higher CFU count observed after 8 seconds of exposure at $60\,^{\circ }\hbox {C}$ than after 6 seconds. However, the difference was not significant. The calculated D-value at $t_{set}=6\,s$ and $t_{set}=8\,\textrm{s}$ where therefore $D_{6\,\textrm{s},60\,^\circ C}=10.77\,\textrm{s}$ and $D_{8\,\textrm{s},60\,^\circ C}=16.56\,\textrm{s}$. All thermal inactivations at $60\,^{\circ }\hbox {C}$ were statistically significant $(p_{6\,s}=0.005, p_{8\,s}=0.006, p_{10\,s}=0.001)$. Similar behaviour is observed after 3 and 4 s of set exposure time at $70\,^{\circ }\hbox {C}$. The thermal inactivation after 2 s of set exposure time was statistically not significant $(p_{2\,\textrm{s}}=0.44)$. After 3 s and 4 s the inactivation was significant with $(p_{3\,s}=0.01, p_{4\,s}=0.01)$. When treated for 6 seconds of set exposure time $t_{set}$ at $70\,^{\circ }\hbox {C}$, there was no measurable survival. The differences between all thermally threated probes were not significant.

#### Burkholderia lata

**Fig. 7:**
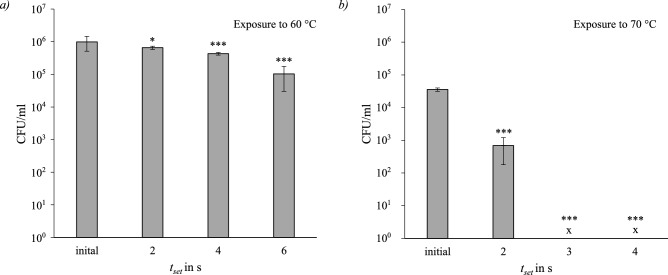
Survival of *Burkholderia lata*; *a* after exposure to $60\,^{\circ }\hbox {C}$ for a set exposure time of (2 s, 4 s, 6 s); *b* after exposure to $70\,^{\circ }\hbox {C}$ for (2 s, 3 s, 4 s). Significance level compared to initial (ANOVA): *$p\le 0.05$; ***$p \le 0.001$.

*Burkholderia lata* was tested at temperatures and set exposure times of $60\,^{\circ }\hbox {C}$ (2 s, 4 s, 6 s), $70\,^{\circ }\hbox {C}$ (2 s, 3 s, 4 s) and $85\,^{\circ }\hbox {C}$ (2 s, 3 s, 4 s). Figure 7 displays the concentration results in the previously used format. As with both previously tested microorganisms, there was no measurable survival at $85\,^{\circ }\hbox {C}$. The initial concentrations at $60^{\circ }\hbox {C}$ and $70^{\circ }\hbox {C}$ differ due to the experiments being conducted on separate days. After exposure times of 3s and 4s at $70\,^{\circ }\hbox {C}$ there was also no measurable activity. The thermal inactivation after all exposure times at $60\,^{\circ }\hbox {C}$ $(p_{2\,s}=0.019, p_{4\,s}=0.001, p_{6\,s}=0.0001)$ and $70\,^{\circ }\hbox {C}$ $(p_{2\,\textrm{s}, 3\,\textrm{s}, 4\,\textrm{s}}<0.0001)$ was significant.

#### MS2

**Fig. 8:**
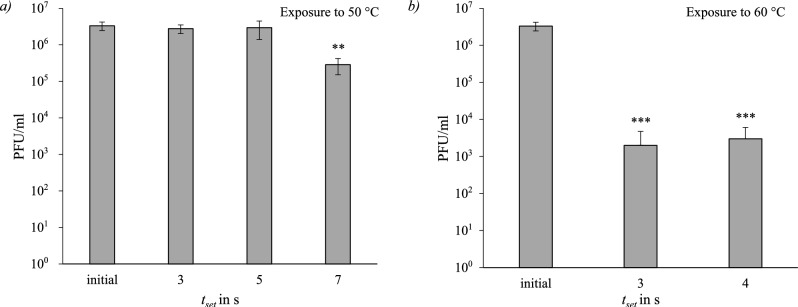
Survival of MS2; *a* after exposure to $50\,^{\circ }\hbox {C}$ for a set exposure time of (3 s, 5 s, 7 s); *b* after exposure to $60\,^{\circ }\hbox {C}$ for (3 s, 4 s). Significance level compared to initial (ANOVA): **$p\le 0.01$; ***$p \le 0.001$.

MS2 was tested at temperatures and set exposure times of $50\,^{\circ }\hbox {C}$ (3 s, 5 s, 7 s), $60\,^{\circ }\hbox {C}$ (3 s, 4 s), $70\,^{\circ }\hbox {C}$ (2 s, 3 s, 4 s) and $85\,^{\circ }\hbox {C}$ (2 s, 3 s). The results are displayed in Plague Forming Units per ml (PFU/ml) in Fig. 8. No measurable survival was detected at any given exposure time after heat treatment at $70\,^{\circ }\hbox {C}$ and $85\,^{\circ }\hbox {C}$. At $50\,^{\circ }\hbox {C}$ there was no measurable inactivation after 3 and 5 seconds. After 7 s exposure time at $50\,^{\circ }\hbox {C}$ the inactivation was 91.4 % with a calculated D-value of $D_{7\,\textrm{s},50\,^\circ C}=5.34\,\textrm{s}$ and significant $(p_{7\,\textrm{s}}=0.01)$. Exposure to $60\,^{\circ }\hbox {C}$ resulted in a statistically significant $(p_{3\,\textrm{s}, 4\,\textrm{s}}<0.0001)$ inactivation of 99.9 % after 3 and 4 seconds with D-values of $D_{3\,\textrm{s},60\,^\circ C}=0.53\,\textrm{s}$ and $D_{4\,\textrm{s},60\,^\circ C}=0.89\,\textrm{s}$.

#### Decimal reduction times and modelling

An average for the decimal reduction time was calculated for the tested temperatures. The results are listed in Table 3 with the according 95 % confidence intervals (CI). As only short periods of thermal inactivation up to 10 s were tested, the confidence interval is larger for higher D-values, such as the thermal inactivation of MS2 at $50\,^{\circ }\hbox {C}$ or *E. viikkiensis* at $60\,^{\circ }\hbox {C}$, due to the relatively low thermal inactivation and high deviation in the experiments.

**Table 3:** D-Values of the tested microorganisms.

Microorganism	D-Value			
	$50\,^{\circ }\hbox {C}$	$60\,^{\circ }\hbox {C}$	$70\,^{\circ }\hbox {C}$	$85\,^{\circ }\hbox {C}$
*Enterococcus viikkiensis*	not tested	$27.7\pm 20.0$	$0.9\pm 0.3$	fully inactivated
*Staphylococcus capitis*	not tested	$12.6\pm 3.2$	$3.8\pm 1.0$	fully inactivated
*Burkholderia lata*	not tested	$5.4\pm 1.7$	$0.4\pm 0.1$	fully inactivated
MS2	$31.1\pm 29.4$	$0.7\pm 0.2$	fully inactivated	fully inactivated

Among all tested microorganisms, bacteriophage MS2 exhibited the highest sensitivity to thermal shock. Due to complete inactivation after the selected exposure periods, it was not possible to determine decimal reduction times (D-values) at 70  $^\circ$C. Among the bacterial strains, *B. lata* was the most susceptible to thermal treatment at both 60  $^\circ$C and 70  $^\circ$C, exhibiting the lowest D-values. At 60  $^\circ$C, *E. viikkiensis* demonstrated the highest thermal resistance, while at 70  $^\circ$C, *S. capitis* exhibited the highest D-value. To predict the temperature-dependent inactivation times of the microorganisms, the reaction rate constant was determined and modeled using the Arrhenius equation (Equation 2). The resulting average activation energy $E_a$ and frequency factor $\ln (a)$ are summarized in Table 4.

**Table 4:** Average activation energy $E_a$ and frequency factor *ln*(*a*) for the tested microorganisms.

Microorganism	Activation Energy $E_a$ in J/mol	Frequency factor *ln*(*a*) in 1/s
*Enterococcus viikkiensis*	302611.3	107.0
*Staphylococcus capitis*	114257.5	39.6
*Burkholderia lata*	239738.5	85.7
MS2	335847.6	122.9

**Fig. 9:**
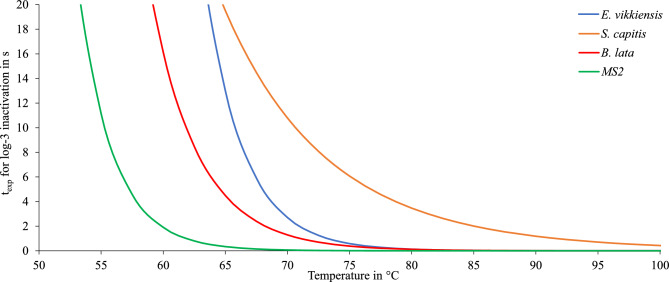
Exposure time for a 3-log inactivation of *E. viikkiensis*, *S. capitis*, *B. lata * and MS2 in dependence of the temperature.

To estimate the required exposure time for a 3-log reduction as a function of temperature, calculations based on the previously described Arrhenius model were performed. The results for the tested microorganisms are shown in Fig. 9. At $85\,^{\circ }\hbox {C}$, all microorganisms except *S. capitis* reach a 3-log inactivation after $\approx 1\,s$ or less.

**Fig. 10:**
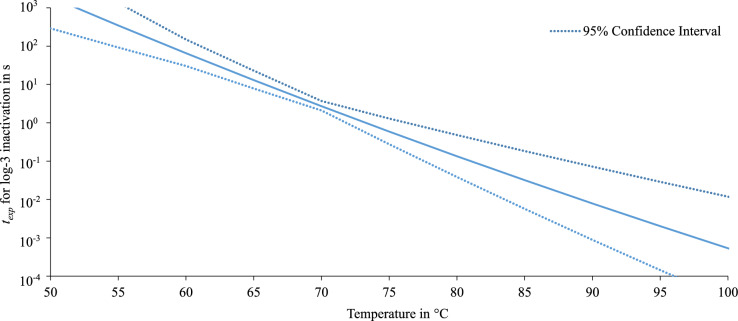
Exposure time for a 3-log inactivation of *E. viikkiensis* in dependence of the temperature with the according 95 % confidence intervall.

Since experimental data were only available for 60 $^\circ$C and 70 $^\circ$C, the predictive accuracy decreases at temperatures outside this range. Therefore, the 95 % confidence intervall was calculated according to the experimental results of *E. viikkiensis* as it had the highest D-value and widest confidence interval at $60\,^{\circ }\hbox {C}$ and *S. capitis* as it had he highest D-value and widest confidence interval at $70\,^{\circ }\hbox {C}$. The results for the thermal inactivation of *E. viikkiensis* including the 95 % confidence interval are shown in Fig. 10. According to the upper bound of the 95 % confidence interval, an exposure time of less than 0.1 s is required to achieve a 3-log reduction at 100 $^\circ$C.

**Fig. 11:**
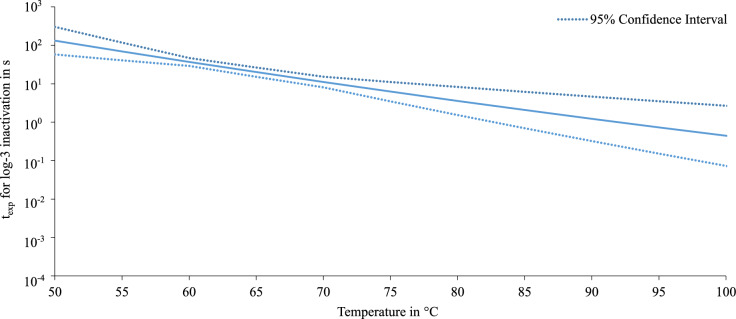
Exposure time for a 3-log inactivation of *S. capitis* in dependence of the temperature with the according 95 % confidence intervall.

The predicted exposure times required for a 3-log reduction of *S. capitis* including the 95 % confidence interval are presented in Fig 11. Similar to *E. viikkiensis*, model predictions for *S. capitis* are based on experimental data obtained at $60\,^{\circ }\hbox {C}$ and $70\,^{\circ }\hbox {C}$. Consequently, the reliability of the extrapolated values decreases at temperatures outside this interval. The 95 % confidence intervall is smaller here, as the data showed less variability. Based on the upper limit of the 95 % confidence interval, an exposure time of approximately 2.7 s is estimated to be sufficient for a 3-log reduction at $100\,^{\circ }\hbox {C}$. However, according to the experimental results no survival was measurable after exposure to $85\,^{\circ }\hbox {C}$ for 2 s.

## Discussion

This study presented an experimental setup for investigating the thermal inactivation of microorganisms through short-duration thermal shocks. The thermal shock was measured using a thermocouple placed in the buffer medium. In the conducted experiments, microorganisms were exposed to a thermal shock with an initial time span of approximately 0.7 s. However, due to non-ideal heat transfer conditions, this duration was corrected from the actual submersion time in the heating bath as detailed in the methods section. To evaluate the resistance of microorganisms to thermal shocks, a literature review was conducted for comparison. Our experimental setup produced significantly shorter exposure times than those typically reported.

For *E. viikkiensis*, no literature data were found regarding resistance to thermal shocks. However, *Enterococcus faecium* is one of the most studied species in this context. Freeman et al.^33^ exposed *E. faecium* in broth culture to 65–80 $^\circ$C, with survival reported up to 20 min at 65–75 $^\circ$C and 3 min at 80  $^\circ$C. The method of heating was not specified. Martinez et al.^34^ showed that the D-values of *E. faecium* at 70 $^\circ$C are dependent on growth temperature and duration. Reported $D_{70}$ values ranged from 0.33 to 1.73 min across growth temperatures of 5–45 $^\circ$C and times from 1 to 1200 h. At 37 $^\circ$C and 24 h growth, a $D_{70}$ of $0.97 \pm 0.06$ min was observed. Heat treatment was performed using a TR-SC thermoresistometer, where inocula were injected into pre-heated buffer with neutral pH. Coe et al.^35^ examined thermal inactivation of *E. faecium* on mash feed, using a heated air stream followed by cooling. A $D_{70}$ value of 135.7 s was reported. In contrast, our study revealed much shorter thermal inactivation times for *E. viikkiensis*, with a $D_{70}$ of $0.9 \pm 0.3$ s. This discrepancy may be due to the intense, short-term thermal shock applied in our experiments, or inherent differences between *E. faecium* and *E. viikkiensis*.

For staphylococci, the potentially vancomycin-resistant *Staphylococcus aureus* has been widely studied. Kennedy et al.^36^ reported D-values between 4.8 and 6.5 min at 60  $^\circ$C in soy broth. Montanari et al.^37^ examined various staphylococcal strains at 80  $^\circ$C. Test tubes were submerged in a water bath, reaching target temperature within 1.5 min. A 6-log reduction in bacterial count occurred within 2–4 min, although survival was observed up to 20 min. In our study, *S. capitis* K1 exhibited significantly shorter inactivation times. The D-values were $12.6 \pm 3.2$ s at 60 $^\circ$C and $3.8 \pm 1.0$ s at 70 $^\circ$C. Complete inactivation was achieved after 2 s treatment time in the 85  $^\circ$C water bath. These differences are likely attributable to the shorter, more intense heat exposure in our setup or species-specific heat resistance.

Only limited data exist regarding thermal inactivation of *Burkholderia* species. No D-values were found in the literature. Schroeder and Loparev^38^ achieved complete inactivation of a high-density suspension ($10^{10}$ CFU/ml) of *Burkholderia cepacia* at 95 $^\circ$C after 45 s using a Denator Stabilizer T1. Perrett and Mawhinney^39^ studied inactivation of *Burkholderia mallei* in equine serum. A suspension ($\ge 10^6$ CFU/ml) was heated to 56  $^\circ$C for up to 30 min. Complete inactivation was observed after 20–30 min, while viable colonies were detected after 10 min. In our study, *B. lata* was the most susceptible bacterium to thermal shocks. D-values were $5.4 \pm 1.7$ s at 60 $^\circ$C and $0.4 \pm 0.1$ s at 70 $^\circ$C. Complete inactivation occurred after 2 s at 85 $^\circ$C.

The MS2 bacteriophage, commonly used as a surrogate for more dangerous viruses such as SARS-CoV-2^26^, was found by Shaffer et al.^40^ to be significantly more heat-resistant than norovirus. They reported D-values of 354.2 min at 50  $^\circ$C, 51.6 min at 60 $^\circ$C, and 14.4 min at 70 $^\circ$C. Heating was performed using a block heater in which the testing tubes reached the target temperature in approximately 2 min. In contrast, our results showed D-values of $31.1 \pm 1.7 \,\textrm{s}$ at 50 $^\circ$C and $0.7\pm 0.2\,\textrm{s}$ at 60 $^\circ$C. No viable MS2 particles were detected after 2 s at 70 $^\circ$C. This suggests MS2 may be particularly susceptible to thermal shocks. Furthermore, Dolan et al.^41^ reported a D-value of 0.64 s at 60  $^\circ$C during microwave treatment of frozen strawberries, though the extent to which microwaves contributed to inactivation is unclear. Grinshpun et al.^42^ studied MS2 inactivation in an airstream and found that exposure times of 0.24–0.33 s at approximately 75 $^\circ$C reduced virus viability by 1-log. In our experiments, no viable MS2 particles were detectable after exposure to 70 $^\circ$C for 2 s.

Compared to literature values, our setup consistently achieved shorter inactivation times across all tested organisms. This is likely due to the rapid application of thermal energy, as opposed to gradual heating and cooling phases used in other studies. The described system is both cost-effective and suitable for replication by other research groups investigating thermal shock inactivation. To test temperatures above $100\,^{\circ }\hbox {C}$, the water could be replaced with a heating bath fluid with a higher boiling point. This change could also improve the heat-up phase if the fluid has a higher thermal conductivity. However the cool-down phase could be extended as the evaporation of the water contributes to cooling down the capillary. Furthermore, immersing the glass capillary horizontally rather than vertically would allow the entire liquid column to be uniformly exposed to the thermal medium. This orientation helps prevent displacement of the liquid column caused by uneven heating and the expansion of air bubbles. For improved modeling and understanding of the underlying mechanisms, further experiments should explore a broader range of temperatures and exposure times. This would also help verify the appropriateness of the log-linear inactivation model used, or suggest alternatives. Especially at lower temperatures the decay curve showed a shoulder effect at short exposure times, which cannot be represented by the model. Extrapolations should be treated with caution, especially outside the tested temperature ranges (50–60 $^\circ$C for MS2 and 60–70 $^\circ$C for other microorganisms), where uncertainty is greater. Nevertheless, results at 85 $^\circ$C indicate that all microorganisms tested experienced $> \log 3$ reductions after less than 0.7 s of corrected or 2 s of actual submersion time.

The collected data can inform the design of public transport vehicle heating systems aimed at achieving thermal inactivation. Specifically, it enables the estimation of relevant parameters such as air volume, exposure duration, and the temperatures required to inactivate potential pathogens. The proposed system is primarily suited for cold climates, where substantial heating capacity is already required. In warmer regions, alternative approaches such as HEPA filtration are likely to be more advantageous.These questions, however, require further investigation to determine whether thermal inactivation of indoor air in public transport vehicles is more energy-efficient or more effective than existing systems. Further research is also necessary to characterize the behavior and thermal response of microorganisms when exposed to heat in aerosolized form. Furthermore, relevant pathogens should also be tested to validate the results obtained with their surrogate organisms. Other potential applications can also be evaluated based on the data obtained. These include the treatment of microbiologically contaminated liquids, such as wastewater, and the thermal inactivation of microorganisms in clinical or healthcare environments. However, the energy efficiency of such approaches must be carefully assessed, as sufficient waste heat is typically not available and active heating of the medium would be required.

## Conclusion

This study investigated thermal inactivation using harmless (BSL-1) surrogates of pathogens and developed an optimized setup in which thermal shocks achieve rapid inactivation. Our findings confirm that significant microbial inactivation can be achieved in short time frames, suggesting that integration into heating, ventilation and air conditioning (HVAC) systems of public transport vehicles is technically viable. At $85\,^{\circ }\hbox {C}$, all tested microorganisms exhibited more than a 3-log reduction after less than 0.7 s of corrected exposure time, corresponding to 2 s of actual submersion. However, the potential integration of such a system, as well as its advantages and limitations compared to existing technologies, still require further investigation. Compared to previous literature values, the application of thermal shocks with a very rapid heating and cooling phase results in faster thermal inactivation. To further assess the applicability of thermal inactivation for aerosolized microorganisms in the cabin air of public transport vehicles, dedicated experiments using aerosolized microorganisms should be conducted. These studies are necessary to verify whether the same inactivation mechanisms and timeframes observed in liquid media also apply under aerosolized conditions. Furthermore also relevant pathogens should be investigated regarding their thermal inactivation characteristics and should be compared to their selected surrogates. While the proposed application focuses on public transport, the findings can be extended to other contexts, such as thermal inactivation in water and wastewater treatment or disinfection strategies in medical and healthcare environments.

## Data Availability

Data is provided within the manuscript. Additional datasets used and analyzed during the current study are available from the corresponding author on reasonable request.
